# Associations of β-Amyloid and Vascular Burden With Rates of Neurodegeneration in Cognitively Normal Members of the 1946 British Birth Cohort

**DOI:** 10.1212/WNL.0000000000200524

**Published:** 2022-07-12

**Authors:** Sarah E. Keuss, William Coath, Jennifer M. Nicholas, Teresa Poole, Josephine Barnes, David M. Cash, Christopher A. Lane, Thomas D. Parker, Ashvini Keshavan, Sarah M. Buchanan, Aaron Z. Wagen, Mathew Storey, Matthew Harris, Ian B. Malone, Carole H. Sudre, Kirsty Lu, Sarah-Naomi James, Rebecca Street, David L. Thomas, John C. Dickson, Heidi Murray-Smith, Andrew Wong, Tamar Freiberger, Sebastian Crutch, Marcus Richards, Nick C. Fox, Jonathan M. Schott

**Affiliations:** From the Dementia Research Centre (S.E.K., W.C., J.M.N., T.P., J.B., D.M.C., C.A.L., A.K. S.M.B., A.Z.W., M.S., M.H., I.B.M., C.H.S., K.L., R.S., H.M.-S, T.F., S.C., N.C.F., J.M.S.), Dementia Research Institute (D.M.C., N.C.F.), Leonard Wolfson Experimental Neurology Centre (D.L.T.), and Department of Brain Repair and Neurorehabilitation (D.L.T.), UCL Queen Square Institute of Neurology; Department of Medical Statistics (J.M.N., T.P.), London School of Hygiene and Tropical Medicine; 4. Department of Medicine (T.D.P.), Division of Brain Sciences, Imperial College London; MRC Unit for Lifelong Health and Ageing at UCL (C.H.S., S.-N.J., A.W., M.R.); Centre for Medical Image Computing (C.H.S.), University College London; School of Biomedical Engineering & Imaging Sciences (C.H.S.), King's College London; and Institute of Nuclear Medicine (J.C.D.), University College London Hospitals, UK.

## Abstract

**Background and Objectives:**

The goals of this work were to quantify the independent and interactive associations of β-amyloid (Aβ) and white matter hyperintensity volume (WMHV), a marker of presumed cerebrovascular disease (CVD), with rates of neurodegeneration and to examine the contributions of *APOE* ε4 and vascular risk measured at different stages of adulthood in cognitively normal members of the 1946 British Birth Cohort.

**Methods:**

Participants underwent brain MRI and florbetapir-Aβ PET as part of Insight 46, an observational population-based study. Changes in whole-brain, ventricular, and hippocampal volume were directly measured from baseline and repeat volumetric T1 MRI with the boundary shift integral. Linear regression was used to test associations with baseline Aβ deposition, baseline WMHV, *APOE* ε4, and office-based Framingham Heart Study Cardiovascular Risk Score (FHS-CVS) and systolic blood pressure (BP) at ages 36, 53, and 69 years.

**Results:**

Three hundred forty-six cognitively normal participants (mean [SD] age at baseline scan 70.5 [0.6] years; 48% female) had high-quality T1 MRI data from both time points (mean [SD] scan interval 2.4 [0.2] years). Being Aβ positive at baseline was associated with 0.87–mL/y faster whole-brain atrophy (95% CI 0.03, 1.72), 0.39–mL/y greater ventricular expansion (95% CI 0.16, 0.64), and 0.016–mL/y faster hippocampal atrophy (95% CI 0.004, 0.027), while each 10-mL additional WMHV at baseline was associated with 1.07–mL/y faster whole-brain atrophy (95% CI 0.47, 1.67), 0.31–mL/y greater ventricular expansion (95% CI 0.13, 0.60), and 0.014–mL/y faster hippocampal atrophy (95% CI 0.006, 0.022). These contributions were independent, and there was no evidence that Aβ and WMHV interacted in their effects. There were no independent associations of *APOE* ε4 with rates of neurodegeneration after adjustment for Aβ status and WMHV, no clear relationships between FHS-CVS or systolic BP and rates of neurodegeneration when assessed across the whole sample, and no evidence that FHS-CVS or systolic BP acted synergistically with Aβ.

**Discussion:**

Aβ and presumed CVD have distinct and additive effects on rates of neurodegeneration in cognitively normal elderly. These findings have implications for the use of MRI measures as biomarkers of neurodegeneration and emphasize the importance of risk management and early intervention targeting both pathways.

Structural MRI measures are widely used as biomarkers of neurodegeneration in Alzheimer disease (AD). Reduced brain volumes on MRI are included in the AT(N) framework for classifying individuals on the AD continuum,^1^ and rates of atrophy quantified from MRI are often used as outcomes in trials, with the expectation that an effective disease-modifying therapy for AD should attenuate rates of atrophy in treated patients relative to controls. Understanding the factors that influence the progression of neurodegeneration is therefore important, particularly in cognitively normal older adults, who are a key target population for strategies aimed at preventing dementia.

Cerebral β-amyloid (Aβ) deposition is an early pathologic feature of AD, emerging up to 20 years before dementia.^2^ It can be quantified with PET and is increasingly used to identify individuals in the preclinical phase of AD who might be eligible for secondary prevention trials.^3^ White matter hyperintensity (WMH) volume (WMHV), a marker of presumed cerebrovascular disease (CVD), is increased among individuals at risk of AD and predicts AD dementia, raising the possibility that CVD may be involved in AD pathogenesis.^4^ There is therefore considerable interest in understanding the relationship between Aβ and WMHs in cognitively normal elderly and whether they act separately or together to influence downstream processes such as neurodegeneration.

The MRC National Survey of Health and Development (NSHD; the 1946 British Birth Cohort) is the world's longest continuously running birth cohort.^5^ Members are virtually identical in age and have been studied extensively since birth, resulting in years of prospectively collected data from across the life course. Now in their 70s and predominantly still dementia-free, a sample have undergone Aβ PET and serial MRI as part of the Insight 46 substudy.^6^ With this unique dataset, the primary aim of this study was to assess whether baseline Aβ deposition and WMHV were associated with rates of neurodegeneration quantified from MRI and, if so, whether their effects were independent or interactive. A further aim was to investigate the contributions of *APOE* ε4 and vascular risk measured at different stages of adulthood to progression of neurodegeneration in later life.

## Methods

Eligibility criteria have been described elsewhere.^7^ In brief, 502 participants were recruited from the NSHD (n = 2,689 at age 69 years), prioritizing members with relevant life-course data, who took part in a study visit at age 60 to 64 years, and who previously indicated that they would be willing to consider attending a study visit in London. Baseline assessments were performed at University College London between May 2015 and January 2018, and follow-up visits were completed between January 2018 and January 2021.

### Standard Protocol Approvals, Registrations, and Patient Consents

Ethics approval was granted by the National Research Ethics Service Committee (REC reference 14/LO/1173), and all participants provided written informed consent.

### Imaging Variables

Participants were scanned on a single Biograph mMR 3T PET/MRI (Siemens Healthcare). They were injected intravenously with 370 MBq of the ^18^F Aβ PET ligand florbetapir at the start of the imaging session, and dynamic data were obtained over 60 minutes. MRI data were acquired simultaneously, including volumetric T1, T2, and fluid-attenuated inversion recovery (FLAIR). Further information on the imaging protocol is reported elsewhere.^6^

Baseline Aβ PET data were processed with an in-house pipeline including pseudo-CT attenuation correction.^8^ Baseline global standardized uptake value ratios (SUVRs) were generated from a composite cortical region of interest, on the basis of a composite that has been evaluated previously,^9^ and an eroded subcortical white matter reference region. Baseline Aβ status was determined by applying a gaussian mixture model to global SUVRs and then taking the 99th percentile of the lower gaussian as the cut point for positivity (eFigure 1, links.lww.com/WNL/B942). SUVRs and Aβ status were also calculated with a whole-cerebellum reference region (see Sensitivity Analyses). Further information on PET methods is provided in eMethods 1.

T1, T2, and FLAIR MRI underwent correction for gradient nonlinearity,^10^ followed by brain-masked N4-bias correction^11^ and visual inspection of image quality. Baseline WMHV was measured by applying an unsupervised automated algorithm, Bayesian Model Selection, to T1 and FLAIR images, generating a global WMHV, which included subcortical gray matter but not infratentorial regions.^12^ Baseline total intracranial volume (TIV) was calculated with the tissues utility in Statistical Parametric Mapping 12.^13^

Changes in whole-brain, ventricular, and hippocampal volume were calculated from baseline and repeat T1 MRI with the boundary shift integral (BSI).^14^ Specifically, the k-means–normalized BSI was used to calculate whole-brain atrophy after affine registration of scan pairs and differential bias correction (DBC).^15^ Ventricular expansion was determined with affine whole-brain registration, followed by an additional rigid registration using the ventricle regions only and calculation of BSI without DBC. Hippocampal atrophy was assessed with affine whole-brain registration, followed by an additional rigid registration focusing on the hippocampus and surrounding regions, with DBC and calculation of BSI with a double-intensity window approach.^16^ Total hippocampal BSI was calculated as the sum of left and right. All registered scan pairs were reviewed to check for longitudinal continuity.

### *APOE* ε4 and Life Course Variables

*APOE* genotyping was performed as described elsewhere,^17^ and participants were defined as ε4 carriers or noncarriers. Resting systolic blood pressure (BP) and an office-based Framingham Heart Study Cardiovascular Risk Score (FHS-CVS) were determined at ages 36, 53, and 69 years, as previously described.^18,19^ The FHS-CVS, which provides a 10-year risk of cardiovascular events, is a weighted sum of age, sex, systolic BP, antihypertensive medication (yes/no), diabetic status (yes/no), current smoking status (yes/no), and body mass index (BMI).^20^ Socioeconomic position was derived from occupation at age 53 years and categorized as manual or nonmanual professions. Smoking status was obtained from a questionnaire at age 68 years (or if missing, a questionnaire at age 60–64 years). BMI was calculated from measurements at the first Insight 46 visit with the formula BMI = kg/m^2^. Diabetes was determined from self-reported history before age 69 years, a glycated hemoglobin level ≥6.5% (equivalent to ≥48 mmol/mol) at age 69 years or being on diabetic medication at the first Insight 46 visit.

### Statistical Analysis

Participants were excluded from analyses if they had dementia, mild cognitive impairment (MCI), or confounding brain disorders at baseline or if they did not have high-quality longitudinal T1 MRI data. Dementia and MCI were defined as described previously.^21^ For analyses involving Aβ or WMHV, participants needed baseline Aβ PET or WMH segmentation data that passed quality control; for *APOE* ε4 analyses, genotype data also were required; and for vascular risk analyses, they needed FHS-CVS or systolic BP data and covariate data at ≥1 time points. A full breakdown of participants is provided as a flowchart (eFigure 2, links.lww.com/WNL/B942).

Group differences were assessed with *t* tests for continuous normally distributed variables, Wilcoxon rank-sum tests for continuous nonnormally distributed variables, and χ^2^ tests for categorical variables. The Spearman rank correlation was used to test associations involving continuous nonnormally distributed variables.

Linear regression was used to test associations between predictors of interest and rates of change in whole-brain, ventricular, and total hippocampal volume. Each model included the BSI measure of interest in milliliters as the outcome, scan interval in years as the explanatory variable, and interactions between scan interval and (1) each predictor variable of interest and (2) each covariate to estimate their association with rate of change. Interactions between 2 variables were tested by including a term for the 3-way interaction between each predictor and scan interval. No constant term was included because the model estimates mean change over time. A more detailed explanation of models used is provided in eMethods 2 (links.lww.com/WNL/B942).

Effects of baseline Aβ and WMHV on each volume change measure were examined in separate models and then together as predictors in a single model, with adjustment for sex, age at baseline scan, and TIV. Baseline Aβ was considered in separate models as a binary Aβ status (positive/negative) and with the continuous global Aβ SUVR measure. Semipartial *r*^2^ values were calculated to assess the relative explanatory contribution of Aβ and WMHV, above the explanatory contribution of all other variables in the model (eMethods 2, links.lww.com/WNL/B942). If relationships were detected with hippocampal atrophy rates, whole-brain BSI was added as a covariate to examine whether effects on hippocampi were disproportionate to global changes. Further models examined whether there was an interaction between Aβ and WMHV and whether there was an interaction between sex and each of Aβ and WMHV.

Separate models were fitted to assess the effects of *APOE* ε4 (carrier/noncarrier) on each volume change measure. Models were initially adjusted for sex, age at baseline scan, and TIV before further adjustment for baseline Aβ status or WMHV to assess whether effects were attenuated after adjustment for these variables. Similar models were fitted to test the contributions of FHS-CVS and systolic BP at each time point (ages 36, 53, and 69 years) to each volume change measure. Because FHS-CVS and systolic BP were associated with WMHV but not with Aβ status in previous Insight 46 analyses,^18,19^ models were initially adjusted for sex, age at baseline scan, TIV, baseline Aβ status, *APOE* ε4 status, and adult socioeconomic position before further adjustment for baseline WMHV to assess whether it might explain any effects. Models with systolic BP as a predictor were also adjusted for smoking status, presence of diabetes, and BMI around the time of the baseline scan. Interactions between Aβ status and each of FHS-CVS and systolic BP at age 69 years were also tested.

Analyses were conducted in Stata 16 (StataCorp, College Station, TX). Regression assumptions were checked by examination of residual plots. If assumptions were not fully met, bootstrapping (2,000 replications) was used to produce bias-corrected and accelerated 95% CIs. Nonlinear associations were assessed with plots of residuals against each predictor and were formally tested by adding quadratic terms to models. Scatterplots of key relationships are shown in eFigure 3 (links.lww.com/WNL/B942).

Sensitivity analyses were also performed to examine the effect of (1) not adjusting for age at baseline scan (healthier individuals may have been more likely to attend at the start of the study, so age might act as a proxy for recruitment bias) and (2) using the whole cerebellum rather than white matter as a reference region for SUVRs (WMHs might influence Aβ PET tracer uptake,^22,23^ which could confound the results of our analyses).

### Data Availability

Anonymized data are available on request.^24^

## Results

Three hundred forty-six participants (mean [SD] age at baseline scan 70.5 [0.6] years; 48% female) had MRI data from both time points (mean [SD] scan interval 2.4 [0.2] years) and were free of dementia, MCI, and confounding brain disorders at baseline.

Median baseline Aβ SUVR was 0.54 (interquartile range 0.51–0.58), and 16.7% of participants were classified as Aβ positive (SUVR cut point for positivity 0.61). Median baseline WMHV was 2.7 mL (interquartile range 1.5–6.1 mL) and did not differ significantly by Aβ status (*z* = −0.667; *p* = 0.50). There was, however, a weak positive correlation between baseline WMHV and Aβ SUVR (*r* = 0.16; *p* < 0.01). Age at baseline scan was not significantly associated with baseline WMHV (*r* = 0.08; *p* = 0.12), Aβ SUVR (*r* = −0.01; *p* = 0.82), or Aβ status (t [339] = 0.7032; *p* = 0.48), and there were no significant sex differences in baseline WMHV (*z* = −1.533; *p* = 0.13), Aβ SUVR (*z* = 0.637; *p* = 0.52), or Aβ status (χ^2^ = 0.5617; *p* = 0.45). Further characteristics are summarized in Table 1, together with those of the full Insight 46 sample for comparison.

**Table 1:**
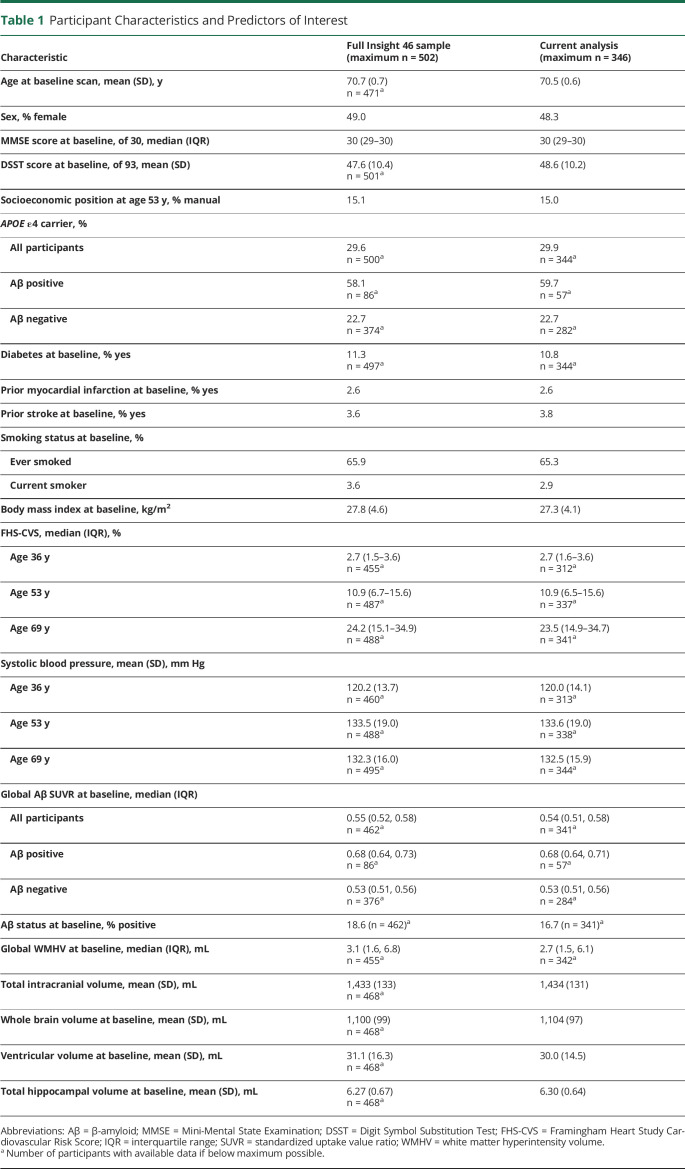
Participant Characteristics and Predictors of Interest

Mean rates of whole-brain and hippocampal atrophy were 5.86 and 0.039 mL/y (equivalent to 0.5%/y and 0.6%/y), and mean rate of ventricular expansion was 1.24 mL/y (Table 2). There were no significant sex differences in rates of neurodegeneration after controlling for TIV, but there were associations with age. Specifically, older age at baseline scan was related to significantly greater rates of ventricular expansion (0.16 mL/y faster per 1-year increment in age; 95% CI 0.03, 0.30) and hippocampal atrophy (0.009 mL/y faster per 1-year increment in age; 95% CI 0.002, 0.016), and there was a directionally consistent but nonsignificant association with rates of whole-brain atrophy (0.46 mL/y faster per 1-year increment in age; 95% CI −0.04, 0.95).

**Table 2:**
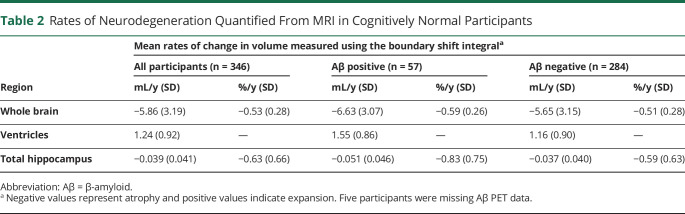
Rates of Neurodegeneration Quantified From MRI in Cognitively Normal Participants

### Effects of Baseline Aβ Deposition

**Figure 1:**
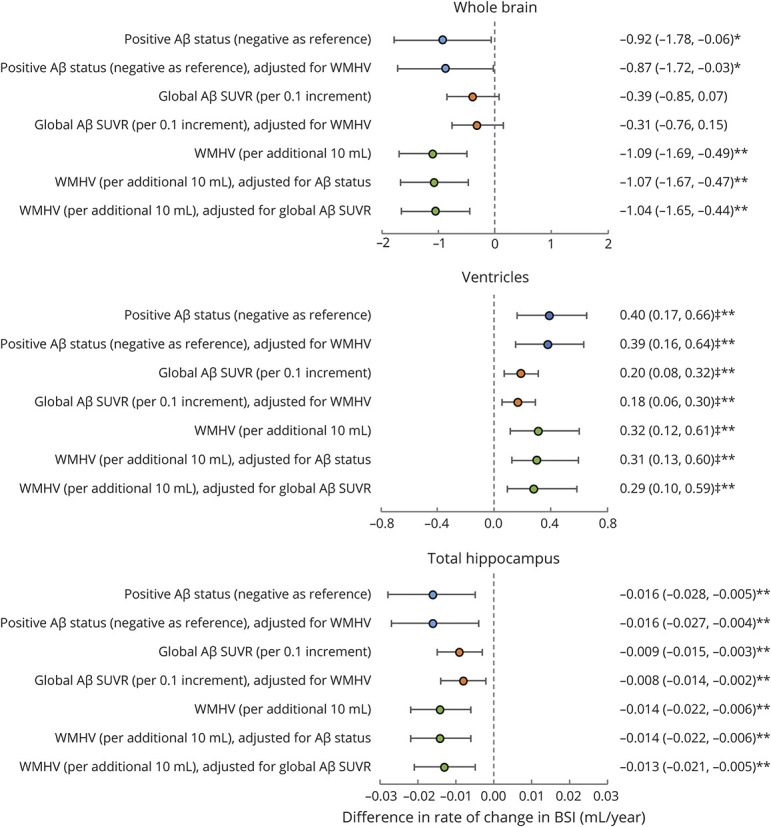
Forest Plot Showing Coefficients and 95% CIs for Associations of Baseline Aβ and WMHV With Rates of Neurodegeneration Quantified From MRI in Cognitively Normal Participants Aβ = β- amyloid; BSI = boundary shift integral; SUVR = standardized uptake value ratio; WMHV = white matter hyperintensity volume. All models were adjusted for sex, age at baseline scan, and total intracranial volume. *p ≤ 0.05; **p ≤ 0.01. ‡Bias-corrected and accelerated bootstrap 95% CIs.

Results of analyses testing associations of Aβ at age 70 years with subsequent rates of neurodegeneration over the next 2.4 years are provided in Figure 1.

Being Aβ positive (compared to negative) was associated with significantly greater rates of neurodegeneration: 0.92–mL/y faster whole-brain atrophy, 0.40–mL/y greater ventricular expansion, and 0.016–mL/y faster hippocampal atrophy. Similar relationships were seen with higher baseline Aβ SUVR, which had significant associations with greater rates of ventricular expansion (0.20 mL/y faster per 0.1 increment in SUVR) and hippocampal atrophy (0.009 mL/y faster per 0.1 increment in SUVR) and a nonsignificant association with rates of whole-brain atrophy (0.39 mL/y per 0.1 increment in SUVR). There was no evidence of nonlinear associations.

There was an interaction between Aβ and sex whereby Aβ had a greater effect on rate of whole-brain atrophy in women than men (eTable 1, links.lww.com/WNL/B942). Post hoc stratification by sex revealed that being Aβ positive at baseline (compared to negative) was associated with 1.82–mL/y greater atrophy in women (95% CI 0.64 to 3.00) and 0.31–mL/y faster atrophy in men (95% CI −0.93 to 1.56), while a 0.1 increment in baseline Aβ SUVR was associated with 0.85–mL/y greater atrophy in women (95% CI 0.24 to 1.47) and 0.06 mL/y faster atrophy in men (95% CI −0.62, 0.73).

### Effects of Baseline WMHV

Results of analyses testing associations of WMHV at age 70 years with rates of neurodegeneration over the next 2.4 years are provided in Figure 1.

Higher WMHV was associated with significantly greater rates of neurodegeneration: each 10-mL additional WMHV was related to 1.09–mL/y faster whole-brain atrophy, 0.32–mL/y greater ventricular expansion, and 0.014–mL/y faster hippocampal atrophy. There was no evidence of nonlinear associations.

There were no interactions between WMHV and sex (*p* > 0.1, all tests; eTable 1, links.lww.com/WNL/B942).

### Disproportionate Hippocampal Atrophy

Associations of baseline Aβ and WMHV with rates of hippocampal atrophy were attenuated after adjustment for whole-brain atrophy such that only the effect of Aβ SUVR remained significant. Specifically, each 0.1 increment in Aβ SUVR was associated with 0.005–mL/y faster hippocampal atrophy (95% CI 0.000 to 0.010), while Aβ positivity was associated with 0.008–mL/y greater hippocampal atrophy (95% CI −0.001 to 0.017), and each 10 mL additional WMHV was associated with 0.005–mL/y faster hippocampal atrophy (95% CI −0.001 to 0.012).

### Independent and Interactive Effects of Baseline Aβ Deposition and WMHV

Effects of Aβ and WMHV on rates of neurodegeneration remained similar when they were assessed together as predictors in the same model (Figure 1).

After WMHV was accounted for, in addition to age, sex, and TIV, Aβ status explained an additional 1.1% of the variance in whole-brain atrophy rate, 2.6% of the variance in ventricular expansion rate, and 2.1% of the variance in hippocampal atrophy rate. After Aβ status was accounted for, in addition to age, sex, and TIV, WMHV explained an additional 3.3% of the variance in whole-brain atrophy and ventricular expansion rate and 3.1% of the variance in hippocampal atrophy rate.

There were no interactive effects of Aβ and WMHV (*p* > 0.1, all tests; eTable 1, links.lww.com/WNL/B942).

### Effects of *APOE* ε4 Status

Results of analyses testing associations of *APOE* ε4 with rates of neurodegeneration around age 70 years are reported in Table 3.

**Table 3:**
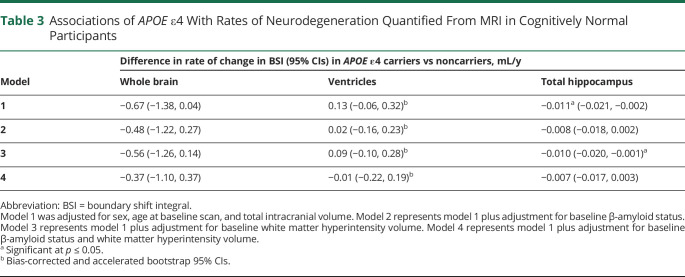
Associations of *APOE* ε4 With Rates of Neurodegeneration Quantified From MRI in Cognitively Normal Participants

*APOE* ε4 carriers had significantly greater rates of hippocampal atrophy (0.011 mL/y faster than noncarriers), and there were directionally consistent but nonsignificant relationships with rates of whole-brain atrophy (0.67 mL/y higher than noncarriers) and ventricular expansion (0.13 mL/y faster than noncarriers). Effects were attenuated after adjustment for Aβ status and, to a lesser extent, WMHV.

### Effects of Exposure to Vascular Risk at Ages 36, 53, and 69 Years

Results of analyses testing associations of FHS-CVS and systolic BP at different stages of adulthood with rates of neurodegeneration around age 70 years are reported in Tables 4 and 5, respectively.

**Table 4:**
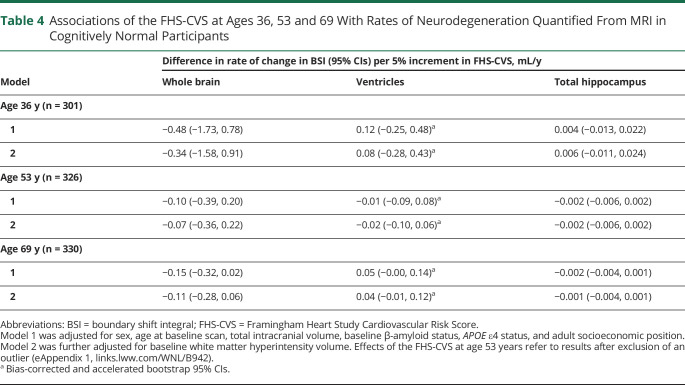
Associations of the FHS-CVS at Ages 36, 53 and 69 With Rates of Neurodegeneration Quantified From MRI in Cognitively Normal Participants

**Table 5:**
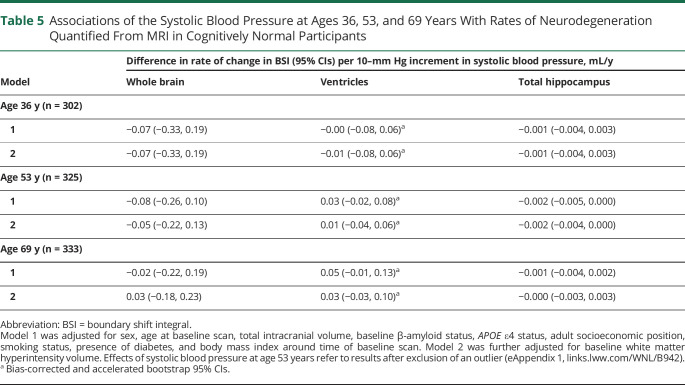
Associations of the Systolic Blood Pressure at Ages 36, 53, and 69 Years With Rates of Neurodegeneration Quantified From MRI in Cognitively Normal Participants

Higher FHS-CVS and systolic BP at age 53 years were initially found to be associated with faster rates of hippocampal atrophy in later life, but effects were small and no longer significant after exclusion of an influential data point (eAppendix 1, links.lww.com/WNL/B942). Otherwise, there were no significant relationships of FHS-CVS or systolic BP with rates of neurodegeneration when assessed across the whole sample and no evidence of nonlinear associations.

Systolic BP at age 69 years did not interact with Aβ status, but there were differential effects of FHS-CVS at age 69 years by Aβ status (eTable 1, links.lww.com/WNL/B942). Specifically, higher FHS-CVS at age 69 was related to significantly greater rates of whole-brain atrophy (0.20 mL/y faster per 5% increment in FHS-CVS; 95% CI 0.01, 0.40) and hippocampal atrophy (0.004 mL/y faster per 5% increment in FHS-CVS; 95% CI 0.001, 0.006) in Aβ-negative individuals, whereas it had nonsignificant and directionally opposite effects on rates of whole-brain atrophy (0.05 mL/y slower per 5% increment in FHS-CVS; 95% CI −0.32, 0.41) and hippocampal atrophy (0.004 mL/y slower per 5% increment in FHS-CVS; 95% CI −0.001, 0.010) in Aβ-positive individuals. There was a substantial difference in sample size between groups, however, with considerably fewer Aβ-positive (n = 56) than Aβ-negative (n = 274) participants.

### Sensitivity Analyses

Rerunning analyses without adjustment for age at baseline scan did not substantially change our findings (eAppendix 2, links.lww.com/WNL/B942). After analyses were rerun with Aβ SUVRs with a whole-cerebellum reference region, slightly fewer participants (15.8% vs 16.7%) were Aβ positive (SUVR cut point for positivity 1.08); associations between baseline Aβ status and rates of neurodegeneration were similar while relationships between baseline Aβ SUVR and rates of neurodegeneration were somewhat weaker but directionally the same; there was a reduced correlation between baseline Aβ SUVR and WMHV (*r* = 0.02; *p* = 0.75); and the interactions between Aβ and sex and between Aβ and the FHS-CVS at age 69 years were decreased and nonsignificant (eAppendix 3).

## Discussion

In this population-based sample of cognitively normal elderly of almost identical ages, we examined predictors of rates of neurodegeneration. Key findings were that both Aβ positivity and higher WMHV were related to faster rates of whole-brain atrophy, ventricular expansion, and hippocampal atrophy, and their effects were independent.

WMHs have a heterogeneous etiology and underlying pathology, but in older adults, they are largely thought to occur as a result of chronic ischemia due to small vessel disease (SVD).^25^ We found no difference in WMHV between Aβ-positive and -negative participants, but there was a weak positive correlation between WMHV and Aβ SUVR defined using a white matter reference region. This relationship was no longer observed using SUVRs with a whole-cerebellum reference region, raising the possibility that quantification of Aβ using a white matter reference region may be influenced by the presence of WMHs or SVD, as has been described previously.^22,23^

Prior studies investigating the relationship between WMHs and Aβ on PET have reported mixed results, but a recent systematic review concluded that they were mostly independent processes.^26^ Some studies have observed increased WMHV in relation to lower Aβ_42_ in CSF,^27-29^ while others have not or have only found a relationship in individuals with AD dementia rather than in people with MCI or healthy controls.^30-32^ There is also evidence that individuals with cerebral amyloid angiopathy, a form of SVD involving deposition of Aβ in blood vessels, have greater WMHs with a predilection for posterior brain regions.^33,34^ This may explain some of the variability in findings between studies.

Regardless of the reference region used to calculate Aβ SUVR, we observed that both Aβ positivity and greater WMHV were independently associated with faster rates of whole-brain atrophy, ventricular expansion, and hippocampal atrophy. The relative contribution of WMHV was somewhat greater than that of Aβ, and there was no evidence that Aβ and WMHV interacted in their effects. These findings are supportive of the hypothesis that Aβ and CVD act predominantly via distinct rather than synergistic pathways and are consistent with a number of other studies that have shown independent effects in relation to rates of atrophy or cognitive decline.^32,35-37^

It is notable that Aβ-positive (versus Aβ-negative) individuals had ≈15% faster whole-brain atrophy and 50% greater hippocampal atrophy rates, and higher Aβ SUVR was associated with disproportionate progressive hippocampal atrophy, despite participants being cognitively normal and years before significant numbers are expected to develop dementia. The effect of WMHV on disproportionate hippocampal atrophy was directionally similar but nonsignificant. Selective vulnerability of the hippocampus is a characteristic feature of early AD, but it has also recently been reported in relation to WMHs, including in healthy controls.^38^

We initially observed an interactive effect of Aβ and sex whereby higher Aβ deposition at baseline was associated with faster rates of whole-brain atrophy in women but not in men, suggesting that women are perhaps more susceptible to the consequences of Aβ. Women are known to be at higher risk of AD, which may be related partly to their longer lifespan, but sex differences in relationships between AD pathologies or risk factors and downstream atrophy or cognition have been reported.^39,40^ The interaction we observed between Aβ and sex was reduced and nonsignificant, however, using SUVRs with a whole cerebellum reference region, which could mean that our original finding was a spurious result or that methods of Aβ measurement may be affected by sex differences in some way.

There were no clear associations between FHS-CVS or systolic BP (at ages 36, 53 and 69 years) and progressive neurodegeneration in later life when assessed across the whole sample. We previously demonstrated that higher FHS-CVS or BP, particularly in early or middle adulthood, was related to smaller brain volumes at age 70 years.^18,19^ This likely reflects that cross-sectional volumes are more indicative of the effects of brain insult(s) before the point of brain imaging and that vascular risk exposure earlier in life is perhaps more detrimental to brain health or associated with greater cumulative risk exposure. We also considered whether differences in findings might be related to a reduction in statistical power because there were fewer participants with longitudinal data. However, post hoc analyses using the smaller sample of this study showed similar relationships with cross-sectional volumes to those previously reported (data not shown).

There was also no evidence that vascular risk and Aβ acted synergistically to influence rates of neurodegeneration, which contrasts with findings from other studies that examined their effects on tau deposition and cognitive decline.^41,42^

The interpretation of the association between older age at baseline scan (despite the narrow age range of the sample) and faster rates of neurodegeneration is uncertain. Previous studies have demonstrated that rates of ventricular expansion are relatively stable before the age of 70 years at ≈1 mL/y but accelerate thereafter, approaching 4 mL/y toward the age of 80 years.^43,44^ This is comparable to the effect we observed (0.16 mL/y faster per 1-year increment in age). Alternatively, age effects in Insight 46 might reflect a degree of recruitment bias in that healthier individuals may have been more likely to attend at the start of the study. While there was no evidence of this in a previous analysis looking at self-reported health and disease burden in Insight 46,^7^ untested differences may still exist.

The findings of this study have implications for the use of MRI measures as biomarkers of neurodegeneration in AD. The AT(N) framework proposes the use of biomarkers of Aβ (A), tau (T), and neurodegeneration (N) to classify individuals on the AD continuum.^1^ However, neurodegeneration is not specific to AD, and this study highlights that CVD, as represented by WMHs, has significant independent effects on neurodegeneration, which are potentially greater in magnitude than those of Aβ. Thus, the findings of this study are supportive of the view of others^45^ that CVD biomarkers should be added to the AT(N) framework, something that has already been discussed as a possibility in a recent position article.^1^ Our findings also highlight the importance of accounting for CVD in AD trials in which MRI measures are included as outcomes because its presence might confound detection of treatment effects, particularly in the preclinical phase when the relative contribution of CVD may be greater.

The results of this study also have broader relevance to our understanding of the processes leading to dementia. While this was a cognitively normal population, it is reasonable to infer that increased rates of neurodegeneration may have subsequent consequences for cognition, given that they are known to be correlated.^46^ Thus, our findings are in keeping with the idea that Aβ and CVD influence risk of cognitive decline predominantly through distinct pathways and that CVD does not contribute to the development of AD pathology per se but may act by lowering the threshold for onset of dementia. Early interventions and risk management targeting both potential pathways are therefore likely to be important. The attenuation of the association between *APOE* ε4 and rates of neurodegeneration after adjustment for Aβ and WMHV suggests that the effects of *APOE* ε4 were mediated by Aβ and, to a lesser degree, WMHV. This is consistent with *APOE* ε4 primarily, but not exclusively, influencing risk of dementia via Aβ deposition.^47^

Key strengths of this study include its population-based setting and prospectively collected data. Participants were also almost identical in age and underwent imaging on a single scanner using a standardized protocol. This is reflected in their mean (SD) atrophy rates (whole brain 5.86 [3.19] mL/y; hippocampal 0.039 [0.041] mL/y), which were considerably less variable (SD/mean ratios around half) compared to those reported by the Alzheimer's Disease Neuroimaging Initiative (whole brain 6.27 [6.15] mL/y; hippocampal 0.052 [0.089] mL/y) and Australian Imaging and Biomarker Lifestyle study (whole brain 5.46 [7.0] mL/y; hippocampal 0.031 [0.061] mL/y), despite the use of the same BSI measurement technique.^48,49^

As documented before, however, Insight 46 has some limitations in terms of generalizability.^21^ Participants were all White, so findings may not be translatable to more ethnically and culturally diverse populations. They also had marginally higher educational attainment, socioeconomic position, and self-rated health than the larger NSHD sample, which suggests that those with poorer health may have been underrepresented.^7^ Without tau PET, we were unable to fully characterize participants according to the AT(N) framework and could not examine whether tau is more strongly related to neurodegeneration than Aβ or whether it interacts with WMHs, as reported elsewhere.^50,51^ Furthermore, because our analyses were limited to global and hippocampal volume measures, we could not exclude the possibility that Aβ interacts with WMHs or vascular risk to influence rates of neurodegeneration in other brain regions. Last, future analyses will be important to assess how longitudinal changes in Aβ and WMHV relate to rates of neurodegeneration, which may provide further support for the independence of these processes.

In conclusion, Aβ and CVD have distinct and additive effects on rates of neurodegeneration in cognitively normal elderly. These findings have implications for the use of MRI measures as biomarkers of neurodegeneration in the preclinical phase of AD and for understanding the processes that confer increased risk of dementia.

## Glossary

Aβ: β-amyloid
AD: Alzheimer disease
BMI: body mass index
BP: blood pressure
BSI: boundary shift integral
CVD: cerebrovascular disease
DBC: differential bias correction
FHS-CVS: Framingham Heart Study Cardiovascular Risk Score
FLAIR: fluid-attenuated inversion recovery
MCI: mild cognitive impairment
NSHD: National Survey of Health and Development
SUVR: standardized uptake value ratio
SVD: small vessel disease
TIV: total intracranial volume
WMH: white matter hyperintensity
WMHV: WMH volume
